# Determination of Five Coumarins in Angelicae Pubescentis Radix from Different Origins by HPTLC-Scanning

**DOI:** 10.1155/2022/3415938

**Published:** 2022-08-29

**Authors:** Dangtong Ji, Qian Li, Hanting Yang, Yue Fan, Ting Wang, Yuying Chen

**Affiliations:** State Key Laboratory of Aridland Crop Science, College of Agronomy, Gansu Agricultural University, Lanzhou 730070, China

## Abstract

The HPTLC method is widely used in the field of quality evaluation and component analysis of traditional Chinese medicine (TCM). This work developed an HPTLC method to determine the five effective components of osthole, columbianadin, isoimperatorin, oxypeucedanin, and imperatorin in *Angelicae Pubescentis* Radix (APR) from twelve different origins, and the quality difference was analyzed by comprehensive factor analysis and cluster analysis. The results showed that the calibration curves of five components exhibited good linearity within the linear ranges (0.8–4.0 *μ*g). The RSD of precision was 1.06%–1.21%, and the repeatability and stability tests were good. The results of cluster analysis showed that the APR from 12 different areas was divided into two categories, and at the same time, it was found that the quality of Dazhou in Sichuan and Huating in Gansu was better than in other areas. In this study, a simple, rapid, and efficient method for quality evaluation of TCM was established by the HPTLC method.

## 1. Introduction

Angelica Pubescentis Radix (APR) is the dry root of *Angelica pubescens* Maxim. *f*. biserrata Shan et Yuan, which belongs to the Apiaceae family [1]. It mainly grows in China, such as Jiangxi, Hubei, and Sichuan provinces. The root of *Angelica pubescens*, known as the traditional herbal drug Duhuo in Chinese, has been used to treat rheumatism, pain, fever, and heaviness of the low back and knees for thousands of years in China [2]. It was usually excavated in early spring or late autumn to remove fibrous roots and soil, piled up for 2–3 d, and heated to dry until soft. Many chemical constituents of APR have been reported since 1957. The coumarins and volatile oil were its main components [3]. In addition, there were organic acids, sugars, and other components in APR. It has been divided into 94 compounds including 69 coumarins and 25 other compounds from APR [4]. In the past 30 years, numerous studies have shown that the extracts from APR have effect of analgesic, anti-inflammatory, and deworming activity and also showed well effects on the central nervous and cardiovascular system. From the perspective of pharmacological activity and phytochemistry of TCM, coumarins are considered to be the most important active ingredients with a wide range of pharmacological effects [5–7].

As known to us, HPLC, UV spectrophotometry, NIRS, LC-MS, and other technologies are mostly used to analyze and determine the content of chemical components and natural compounds [8–13]. Gao et al. established the quality standard of APR by UV-Vis spectrophotometry combined with TLC [14], and the results showed that TLC separation was effective, which has a certain reference value for the study of effective components of APR. Wang et al. established an HPLC fingerprint analysis method for coumarins in APR [15]. Cluster analysis and HPLC-ESI-MS were used to analyze APR from a qualitative point of view, so as to provide the basis for APR quality evaluation and clinical application. Ge et al. established a method for qualitative and quantitative analysis of chemical components in APR by UHPLC–Q-TOF-MS [16]. It could distinguish and identify APR from different producing origins, and the establishment of this method provided reference and a basis for the qualitative detection and identification of TCM.

There are many kinds of complex chemical components in medicinal plants, and the active components were usually regarded as their index components. The analysis and determination of these active components play an important role in the quality control of medicinal materials. Problems such as high operation cost, complex operation, and time consumption exist in the process of chemical composition qualitative and quantitative analysis. HPTLC was a quantitative analysis method based on TLC, and due to its unique characteristics of low cost, simple operation, high accuracy, and good reproducibility, it has been gradually applied to the identification and measurement of pharmaceutical chemical components [17–20]. The main advantages of HPTLC were reducing the time and cost of sample analysis [21]. HPTLC could simultaneously measure different sample contents on the same thin-layer plate, which makes HPTLC overcome the limit of plate efficiency while maintaining its original advantages [22, 23]. Cai et al. have determined the contents of luteolin, apigenin, and acacia in *Chrysanthemum morifolium* cv. ‘Chuju' by HPTLC [24]. A rapid, simple, and accurate method for drug analysis has been established with HPTLC as quality control. ChP (2020 edition) takes osthole and columbianadin as its main index components for the quality control of APR. However, there were many complex chemical components in APR, and the difference in quality could not be fully reflected by the two chemical components in ChP. In this work, in order to solve the problems in the determination of chemical components, the advantages of HPTLC were used to analyze and determine the chemical components of APR from 12 different origins. It is expected to establish a new method for the determination of chemical components in medicinal materials by HPTLC. It can be used for the rapid detection and identification of the quality of TCM to provide a theoretical basis and technical reference.

## 2. Materials and Methods

### 2.1. Apparatus

Qualitative and quantitative HPTLC Densitometer CD 60 (BS131.800, Biostep GmbH) and three-black-box analysis using UV (ZF-7N, Shanghai Jiapeng Technology Co., Ltd.,) analysis were employed in this study.

### 2.2. Materials and Reagents

Osthole (Lot: 18032005, purity ≥98%), columbianadin (Lot: 19091001, purity ≥98%), isoimperatorin (Lot: 18062202, purity ≥98%), imperatorin (Lot: 18051502, purity ≥98%), and oxypeucedanin (Lot: y27s9s65152, purity ≥98%) were purchased from Chengdu Pufeide Biotechnology Co., Ltd. The following reagents were used: methanol (Lot: 20191120, Sinopharm Chemical Reagent Co., Ltd.), petroleum ether (60–90, Lot: 20200814, China Pharmaceutical Group Chemical Reagent Co., Ltd.), and ethyl acetate (Lot: 20190301, Tianjin Fuyu Fine Chemical Co., Ltd.). Methanol, petroleum ether, and ethyl acetate used in this experiment are all analytically pure.

APR from different places was purchased, which were identified as APR by Professor Yuan Chen, Department of Chinese herbal medicine cultivation and identification, Gansu Agricultural University. The sources of APR from different origins are shown in Table 1.

### 2.3. Methods

#### 2.3.1. Scanning Method of HPTLC

The dried samples of 12 batches of the APR powder (1.00 g) were ultrasonic extracted with 10 mL methanol for 20 min and filtered, respectively.

The osthole, columbianadin, isoimperatorin, oxypeucedanin, and imperatorin reference were dissolved in methanol and prepared into a solution containing 0.4 mg per 1 mL, respectively, as the reference solution.

The standard of osthole, columbianadin, isoimperatorin, oxypeucedanin, and imperatorin control was dissolved with methanol in a 25 mL flask. Reserve solution containing 1000 *μ*g of mixed reference per 1 mL was prepared. The 10 mL reserve liquid accurately measured was placed in a 25 mL volumetric flask, and methanol was added to make a mixed reference solution.

#### 2.3.2. TLC Scanning Conditions

**Figure 1:**
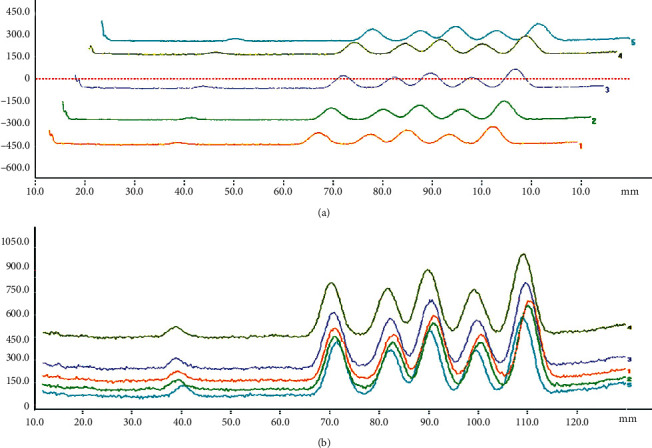
Chromatogram profile of standard and samples. *Note*. (a), (b) 1–5 are HPTLC scanning images of 2, 4, 6, 8, and 10 *μ*L sampling volume of the standard solution and sample solution. The chromatographic peaks from left to right were oxypeucedanin, imperatorin, osthole, columbianadin, and isoimperatorin.

**Figure 2:**
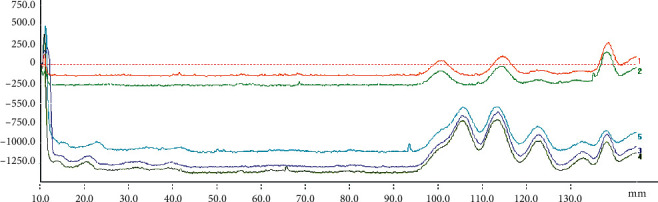
HPTLC 3D diagram of the test solution. *Note*. 1 and 2 are HPTLC scanning images of the mixed standard solution, and 3–5 are HPTLC scanning images of the sample solution. The chromatographic peaks from left to right were oxypeucedanin, imperatorin, osthole, columbianadin, and isoimperatorin.

A thin layer of silica gel plates (100 × 25) was activated in a 110°C electric constant temperature blast drying oven for 30 min and cooled in a dry environment. Twelve different origins of APR sample solutions were spotted in GF_254_ thin-layer silica gel plates (100 × 25) with a sample volume of 6 *μ*L. Petroleum ether (60–90°C)-ethyl acetate (7 : 3) was used as the developing agent. After developing, it was taken out and dried and inspected under the ultraviolet lamp at 365 nm. The test substance showed spots of the same color at the same position as the control substance in the chromatogram. The plate for osthole, columbianadin, isoimperatorin, oxypeucedanin, and imperatorin reference substance and the corresponding spots in the test substance was scanned under wavelength at 365 nm using an HPTLC scanner (Figures 1 and 2). The absorbance integral value of the test sample and the reference sample were measured and calculated.

#### 2.3.3. HPLC Method for Verification

The analytes were separated by a ZORBAX SB-C18 analytical column (250 mm × 4.6 mm, 5 *μ*m). The samples with a volume of 10 *μ*L were injected into the column, and the temperature was maintained at 30°C for the separation. The mobile phase was (A) methanol and (B) water with a ratio of 65 : 35 at a flow rate of 1.0 mL/min. The ultraviolet detection wavelength was set to 325 nm, and the analysis time was 30 min.

The detailed process and results were provided in supporting information. The HPLC chromatograms of the mixed standard solution and APR are shown in Figure S1 (supporting information). The analytical method was validated by the determination of the linearity (Table S1, supporting information), precision, repeatability, stability, and recovery.

## 3. Results and Discussion

### 3.1. Investigation of the Linear Relationship

The standard reference solution prepared under method 2.3 was accurately aspirated, respectively. The sample volume of 2, 4, 6, 8, and 10 *μ*L was respectively applied on the GF_254_ thin-layer silica gel plate under the same conditions, and the imaging scanning was carried out. The standard curve was obtained by linear regression with reference sample volume *X* (*μ*g) as abscissa and peak area score *Y* as ordinate (Table 2). The results showed that osthole, columbianadin, isoimperatorin, oxypeucedanin, and imperatorin have a good linear relationship between the sample size and the peak area with the range of 0.8–4.0 *μ*g.

### 3.2. Precision

The mixed reference solutions of osthole, dihydrocarveol angelic acid ester, isoimperatorin, oxypeucedane, and imperatorin were absorbed accurately with a sample size of 8 *μ*L, and the sample was repeated five times. The samples were scanned by TLC. The RSD values of the five components were 1.06%, 1.06%, 1.21%, 1.19%, and 1.10%, indicating that the precision of the method was well.

### 3.3. Stability

The S1 test solution and the mixed reference solution were crossed pointed samples on the same GF_254_ thin-layer silica gel plate. The samples were determined according to the scanning conditions of TLC and scanned every 30 minutes. The results showed that the peak area scores of five components remained stable within 3 h. The RSD values were 1.34%, 1.45%, 1.13%, 1.11%, and 1.32%, respectively.

### 3.4. Repeatability

The powder of test product S1 was taken, and 6 samples of the test product solution were prepared in parallel according to the method mentioned in 2.3.1. The contents of osthole, columbianadin, isoimperatorin, oxypeucedanin, and imperatorin were determined by TLC scanning. The results showed that the RSD of the five components was 1.91%, 1.83%, 1.96%, 1.50%, and 1.87%, respectively. The results indicated that this method has good repeatability.

### 3.5. Determination of the APR Content in Different Places of Origin

12 batches of samples in APR from different areas were prepared into test solution according to the method mentioned in 2.3. The test solution and mixed standard solution were spotted on the same silica gel thin plate. TLC scanning was performed according to the method mentioned in 2.3. The contents of osthole, columbianadin, isoimperatorin, oxypeucedanin, and imperatorin have been determined (Table 3).

### 3.6. Comprehensive Factor Analysis of APR Content in Different Places of Origin

Five components of APR from different origins were analyzed by principal component analysis (comprehensive index = membership values weight). The larger the composite index, the higher the rank. The eigenvalue of principal component 1 of APR from different origins was greater than 1, and the cumulative contribution rate was 66.28%. Therefore, the matrix value of principal component 1 and the contribution rate of each eigenvalue were extracted to calculate the weight value of each index (Table 4). The comprehensive factors of the APR of different origins were calculated. The results showed that the comprehensive indexes of APR in different origins were 0.8656, 0.6185, 0.8087, 0.0000, 0.7144, 0.5019, 0.8992, 0.6130, 0.6236, 0.5055, 0.2994, and 0.4648, respectively. The comprehensive index of APR content of different origins was S7 > S1 > S3 > S5 > S9 > S2. Based on the analysis results of five coumarins in APR from different origins, the content and quality of APR from S7, S1, S3, S5, and S9 origins were higher. The ranking of membership values and comprehensive indexes is shown in Table 5.

### 3.7. Cluster Analysis

**Figure 3:**
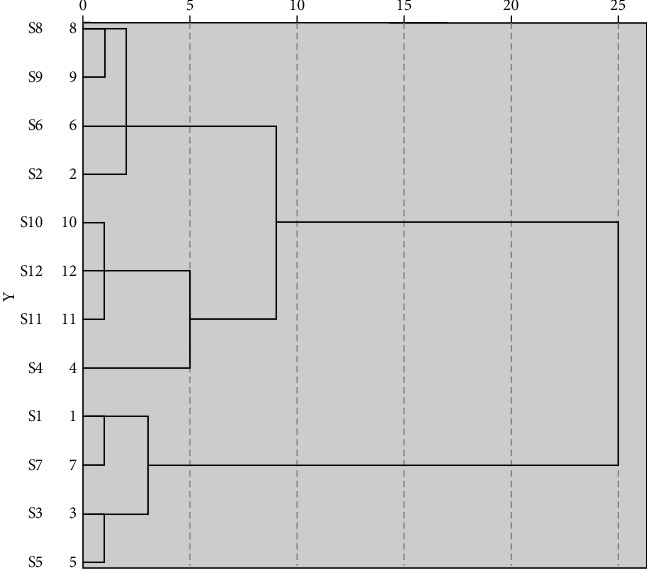
Tree diagram of cluster analysis.

Taking the content of five coumarins in APR from different producing origins as variables, the data were analyzed by cluster analysis with IBM SPSS Statistics software, and the Euclidean Distance was used as the measure. The content analysis of APR from 12 different producing origins is shown in Figure 3.

As shown in Figure 3, when the discrimination distance was less than 25, the samples were divided into two categories. S1, S3, S5, and S7 were clustered into one category. S2, S4, S6, S8, S9, S10, S11, and S12 were clustered into one category. The results indicated that there was a great difference in the quality of APR between the two categories, while there was a small difference in the quality of APR in S1, S3, S5, and S7 production areas. The medicinal materials in S2, S4, S6, S8, S9, S10, S11, and S12 were of similar quality. When the discrimination distance was 5–10, the samples were divided into three categories. S1, S3, S5, and S7 were clustered into one category, S4, S10, S11, and S12 were clustered into one category, and S2, S6, S8, and S9 were clustered into one category. When the discriminant distance was less than 5, the samples were divided into five categories. S2, S6, S8, and S9 were clustered into one category, S10, S11, and S12 were clustered into one category, S1 and S7 were clustered into one category, S3 and S5 were clustered into one category, and S4 was a separate category.

HPTLC is a relatively semiquantitative method for quality evaluation of traditional Chinese medicine, while HPLC can accurately quantitatively analyze the components of traditional Chinese medicine, and its sensitivity and precision are higher than those of HPTLC. In the process of content determination in this study, the HPLC method was used as a reference, which has been provided in supporting information. The two methods showed that the contents of the components were stable between each other. In addition, the comprehensive ranking results showed that there is little difference between the two methods (Table S2, supporting information). Among them, the comprehensive content of S7 was the highest and that of S4 was the lowest, which may be related to the environment and other factors. Compared with HPLC, the HPTLC experiment has more errors in the operation process, including sampling error, expansion error, and detection error. In addition, temperature and other external operating conditions have a great influence on the measurement of some components. Therefore, there are differences between HPTLC and HPLC in the content determination of medicinal materials. This leads to inconsistent measurement results for S5–S7 columbianadin. In summary, the new method has the characteristics of high accuracy and good reproducibility. Compared with HPLC, this method has low cost, simple operation, and reduced the time of sample analysis. The established method provides a new way for rapid analysis and determination of multiple components in medicinal materials.

## 4. Conclusions

In this study, APR from different origins was taken as the research object, and the qualitative and quantitative analysis method of APR was established by using HPTLC. The contents of osthole, columbianadin, isoimperatorin, oxypeucedanin, and imperatorin were calculated, respectively. Furthermore, cluster analysis was used to evaluate the quality differences of APR from different origins. The results showed that HPTLC has the advantage of simplicity, rapidness, and high efficiency in the determination of chemical constituents of TCM. The results of the analysis and determination of five active components in APR were obvious and stable, and the precision, repeatability, and stability of the test were good. This work provides a data reference for the rapid detection and identification of the quality of TCM and clinical medication specification.

## Figures and Tables

**Table 1:** Origin information of Angelicae Pubescentis Radix.

Number	Place of origin
S1	Huating, Gansu
S2	Deshengtang, Gansu
S3	Yunnan
S4	A.pub, Chongqing
S5	AbaPrefecture, Sichuan
S6	Sichuan
S7	Dazhou, Sichuan
S8	Zhirentongji, Sichuan
S9	Anningtaixing, Hubei
S10	Zhongyoujiankang, Hubei
S11	Huirentang, Hubei
S12	Hubei

**Table 2:** Standard curve.

Component	Linear regression equation	Correlation coefficient
Osthole	*Y* = 0.0584*X* + 14.6447	*r* = 0.9922
Columbianadin	*Y* = 0.0411*X* + 10.5898	*r* = 0.9873
Isoimperatorin	*Y* = 0.1943*X* + 179.3765	*r* = 0.9945
Oxypeucedanin	*Y* = 0.1607*X* + 0.5229	*r* = 0.9918
Imperatorin	*Y* = 0.2142*X* + 170.9726	*r* = 0.9935

**Table 3:** Determination of results of APR in different origins (%).

Place of origin	Oxypeucedanin	Imperatorin	Osthole	Columbianadin	Isoimperatorin
S1	0.7589	0.5972	0.6073	0.0822	0.2409
S2	0.5550	0.5568	0.4397	0.0652	0.2688
S3	0.7760	0.6003	0.4860	0.1013	0.1648
S4	0.3312	0.2820	0.2760	0.0288	0.0610
S5	0.8297	0.4683	0.5503	0.0850	0.1340
S6	0.5647	0.4440	0.5137	0.0707	0.0990
S7	0.6500	0.6110	0.5730	0.0990	0.2980
S8	0.5320	0.4150	0.5393	0.0770	0.2630
S9	0.4993	0.4303	0.5617	0.0783	0.2567
S10	0.4283	0.3673	0.5187	0.0807	0.2113
S11	0.3460	0.2827	0.4890	0.0540	0.1857
S12	0.4063	0.3247	0.5093	0.0817	0.2087

**Table 4:** Principal components and weights of APR in different origins.

Component	Pca 1	Weighted value
Oxypeucedanin	0.8103	0.2005
Imperatorin	0.8557	0.2117
Osthole	0.8340	0.2064
Columbianadin	0.9144	0.2262
Isoimperatorin	0.6273	0.1552
Eigen value	3.3140	
Cumulative (%)	66.2812	

**Table 5:** Membership values and composite index rankings of APR in different origins.

Place of origin	Oxypeucedanin	Imperatorin	Osthole	Columbianadin	Isoimperatorin	Comprehensive index	Ranking
S1	0.1720	0.2028	0.2064	0.1666	0.1178	0.8656	2
S2	0.0900	0.1768	0.1020	0.1136	0.1361	0.6185	6
S3	0.1789	0.2048	0.1308	0.2262	0.0680	0.8087	3
S4	0.0000	0.0000	0.0000	0.0000	0.0000	0.0000	12
S5	0.2005	0.1199	0.1709	0.1754	0.0478	0.7144	4
S6	0.0939	0.1042	0.1481	0.1308	0.0249	0.5019	9
S7	0.1282	0.2117	0.1850	0.2191	0.1552	0.8992	1
S8	0.0808	0.0856	0.1640	0.1504	0.1323	0.6130	7
S9	0.0676	0.0954	0.1780	0.1545	0.1282	0.6236	5
S10	0.0391	0.0549	0.1512	0.1620	0.0984	0.5055	8
S11	0.0060	0.0005	0.1327	0.0786	0.0817	0.2994	11
S12	0.0302	0.0275	0.1453	0.1651	0.0967	0.4648	10

## Data Availability

The data used to support the findings of this study are available from the corresponding authors upon request.

## Abbreviations

HPTLC: High performance thin-layer chromatography
TCM: Traditional Chinese medicine
APR: Angelica Pubescentis Radix
UV: Ultraviolet
NIRS: Near infrared spectroscopy
LC-MS: Liquid chromatography mass spectrometry
GC-MS: Gas chromatography mass spectrometry
ChP: Chinese pharmacopoeia
TLC: Thin-layer chromatography.

## Supplementary Materials

Supplementary Materials The chromatographic conditions and results of HPLC. Table S1: standard curve. Table S2: determination of results of APR in different origins (%). Figure S1: HPLC chromatograms of (A) 80 *μ*g/mL mixed standard solution and (B) APR Peak identification as follows: (1) oxypeucedanin, (2) imperatorin, (3) osthole, (4) columbianadin, and (5) isoimperatorin.
