# Supplementary material for: Determination of Five Coumarins in Angelicae Pubescentis Radix from Different Origins by HPTLC-Scanning
Source: J Anal Methods Chem. 2022 Aug 29;2022:3415938. doi: 10.1155/2022/3415938 (PMC9444472; doi:10.1155/2022/3415938)
Supplement: Supplementary Materials — The chromatographic conditions and results of HPLC. Table S1: standard curve. Table S2: determination of results of APR in different origins (%). Figure S1: HPLC chromatograms of (A) 80 μg/mL mixed standard solution and (B) APR Peak identification as follows: (1) oxypeucedanin, (2) imperatorin, (3) osthole, (4) columbianadin, and (5) isoimperatorin. [file 3415938.f1.docx]

**Supporting Information**

**Determination of five coumarins in *Angelicae Pubescentis* Radix from different origins by HPTLC-scanning**

Dangtong Ji, Qian Li *, Hanting Yang, Yue Fan, Ting Wang, Yuying Chen

(Gansu Provincial Key Laboratory of Aridland Crop Science, College of Agronomy, Gansu Agricultural University, Lanzhou 730070，PR China)

*Corresponding author: Qian Li, Email: [liqian1984@gsau.edu.cn](mailto:liqian1984@gsau.edu.cn)

**Supplementary Materials and Methods**

**Chromatographic conditions**

The analytes were separated by a ZORBAX SB-C18 analytical column (250 mm × 4.6 mm, 5 μm). The samples with a volume of 10 μL were injected into the column and the temperature was maintained at 30 ℃ for the separation.

The mobile phase was (A) methanol and (B) water with a ratio of 65:35 at a flow rate of 1.0 mL/min.The ultraviolet detection wavelength was set to 325 nm, the sample analysis time was 30 min.

**Results and Discussion**

**Linearity and precision**

The prepared mixed standard with a concentration of 1000 μg/mL was diluted with methanol to 5, 10, 20, 40, 80 μg/mL. The samples were analyzed by HPLC. The results showed that the five components have a good linear relationship in the determination range (Table 1). The calibration curves covered the range from 5 to 80 μg/mL.

The precision was measured with six repetitions of samples with the same concentration, and the precision was expressed by RSD value. The RSD of each component was 0.08%, 0.06%, 0.18%, 0.05% and 0.11%, respectively, which showed that the precision of the instrument was good.

**Table S1 Standard curve**

| **Component** | **Linear regression equation** | | **Correlation coefficient** | **Linear range (μg/mL)** |
| --- | --- | --- | --- | --- |
| Osthole | Y=4990X+14900 | R^2^=0.9981 | | 5-80 |
| Columbianadin | Y=3630X+8550 | R^2^=0.9965 | | 5-80 |
| Isoimperatorin | Y=4070X+2710 | R^2^=0.9960 | | 5-80 |
| Oxypeucedanin | Y=2800X+12200 | R^2^=0.9946 | | 5-80 |
| Imperatorin | Y=2020X+8170 | R^2^=0.9947 | | 5-80 |

**Repeatability and stability**

The same origin of medicinal materials were performed six times according to the same chromatographic conditions. The contents of osthole, columbianadin, isoimperatorin, oxypeucedanin and imperatorin were 3.37%, 0.53%, 0.46%, 1.24%, 0.30%, and the RSD were 1.24%, 1.25%, 1.34%, 1.14% and 1.17%, respectively.

The first sample was used to characterize stability within 24 h. The results showed that the RSD of the five components was 1.26%,1.41%, 1.65%, 1.47% and 1.51%, respectively. The results demonstrate that the compounds were stable under the detected conditions. The HPLC chromatograms of mixed standard solution and APR are shown in Figure S1.


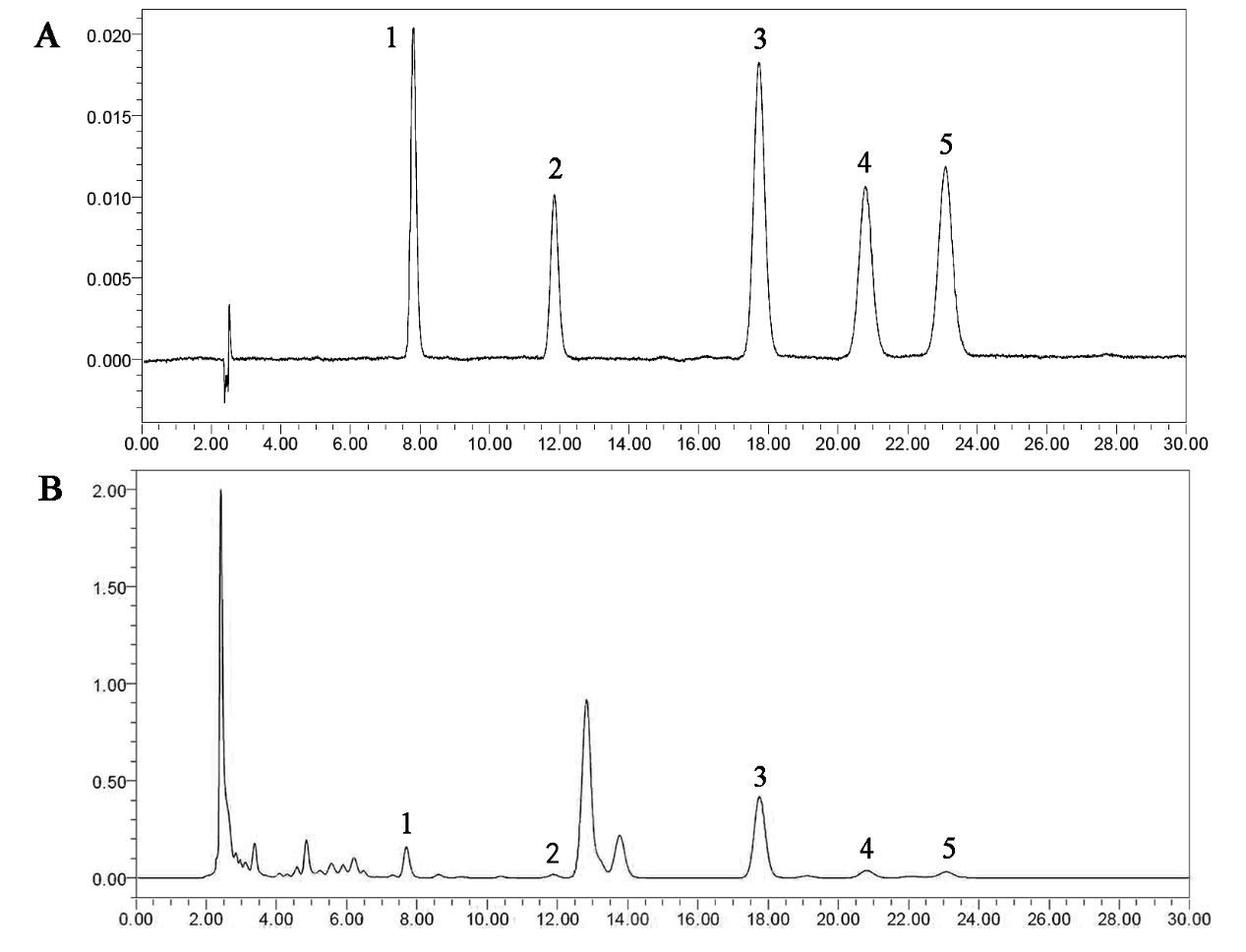


**Figure S1 HPLC chromatograms of (A) 80 μg/mL mixed standard solution and (B) APR Peak identification: (1) oxypeucedanin, (2) imperatorin, (3) osthole, (4) columbianadin, (5) isoimperatorin**

**Recovery**

The sample powder with known content was weighed with about 0.5 g and 6 samples were prepared (S1: Huating,Gansu). An appropriate amount of the mixed reference solution was added, respectively. The powder was detected and analyzed under chromatographic conditions. The recovery of osthole, columbianadin, isoimperatorin, oxypeucedanin and imperatorin were 100.38%, 98.71%, 100.26%, 102.44%, 93.94%, and the RSD were 1.68%, 1.37%, 1.42%, 1.51% and 1.83%, respectively.

**Determination of APR Content by HPLC in Different Places of Origin**

Twelve batches of APR samples from different regions were determined according to chromatographic conditions. The results showed that the linearity of five coumarin components from twelve different habitats was good in the determination range. In addition, the contents of each component were stable (Table S2).

**Table S2 Determination results of APR in different origins (%)**

| **Place of Origin** | **Oxypeucedanin** | **Imperatorin** | **Osthole** | **Columbianadin** | **Isoimperatorin** |
| --- | --- | --- | --- | --- | --- |
| S1  S2  S3  S4  S5  S6  S7  S8  S9  S10  S11  S12 | 1.24  1.28  1.80  1.78  6.71  4.73  7.24  1.61  1.05  1.55  0.99  0.97 | 0.30  0.31  0.03  0.02  0.42  0.07  0.38  0.03  0.01  0.01  0.01  0.02 | 3.37  3.47  3.46  0.35  0.11  0.09  0.04  2.14  2.88  3.28  3.31  2.07 | 0.53  0.54  0.91  0.09  0.02  0.01  0.01  0.57  0.40  0.41  0.77  1.38 | 0.46  0.47  0.16  0.01  0.02  0.01  0.02  0.11  0.13  0.10  0.07  0.17 |
